# Blood Flow and Glucose Metabolism Dissociation in the Putamen Is Predictive of Levodopa Induced Dyskinesia in Parkinson's Disease Patients

**DOI:** 10.3389/fneur.2019.01217

**Published:** 2019-11-21

**Authors:** Maram Aljuaid, Samuel Booth, Douglas E. Hobson, Andrew Borys, Kelly Williams, Audrey Katako, Lawrence Ryner, Andrew L. Goertzen, Ji Hyun Ko

**Affiliations:** ^1^Department of Human Anatomy and Cell Science, Rady Faculty of Health Sciences, Max Rady College of Medicine, University of Manitoba, Winnipeg, MB, Canada; ^2^Neuroscience Research Program, Kleysen Institute for Advanced Medicine, Health Sciences Centre, Winnipeg, MB, Canada; ^3^Section of Neurology, Department of Internal Medicine, Rady Faculty of Health Sciences, Max Rady College of Medicine, University of Manitoba, Winnipeg, MB, Canada; ^4^Department of Radiology, Rady Faculty of Health Sciences, Max Rady College of Medicine, University of Manitoba, Winnipeg, MB, Canada

**Keywords:** cerebral blood flow, glucose metabolism, magnetic resonance imaging, neurovascular coupling hypothesis, positron emission tomography

## Abstract

**Background:** The forefront treatment of Parkinson's disease (PD) is Levodopa. When patients are treated with Levodopa cerebral blood flow is increased while cerebral metabolic rate is decreased in key subcortical regions including the putamen. This phenomenon is especially pronounced in patients with Levodopa-induced dyskinesia (LID).

**Method:** To study the effect of clinically-determined anti-parkinsonian medications, 10 PD patients (5 with LID and 5 without LID) have been scanned with FDG-PET (a probe for glucose metabolism) and perfusion MRI (a probe for cerebral blood flow) both when they are ON and OFF medications. Patients additionally underwent resting state fMRI to detect changes in dopamine-mediated cortico-striatal connectivity. The degree of blood flow-glucose metabolism dissociation was quantified by comparing the FDG-PET and perfusion MRI data.

**Results:** A significant interaction effect (imaging modality × medication; blood flow-glucose metabolism dissociation) has been found in the putamen (*p* = 0.023). *Post-hoc* analysis revealed that anti-parkinsonian medication consistently normalized the pathologically hyper-metabolic state of the putamen while mixed effects were observed in cerebral blood flow changes. This dissociation was especially predominant in patients with LID compared to those without. Unlike the prior study, this differentiation was not observed when cortico-striatal functional connectivity was assessed.

**Conclusion:** We confirmed striatal neurovascular dissociation between FDG-PET and perfusion MRI in response to clinically determined anti-parkinsonian medication. We further proposed a novel analytical method to quantify the degree of dissociation in the putamen using only the ON condition scans, Putamen-to-thalamus Hyper-perfusion/hypo-metabolism Index (PHI), which may have the potential to be used as a biomarker for LID (correctly classifying 8 out 10 patients). For wider use of PHI, a larger validation study is warranted.

## Background

Levodopa (LD) has been the first line of treatment of Parkinson's disease (PD) since its introduction. More than 50% of patients develop Levodopa induced dyskinesia (LID) after 5–10 years of treatment (1). While the exact cause of LID is still unknown, epidemiological studies suggest disease duration, symptom severity, young age at the time of onset, duration of Levodopa treatment and overall dose are the major risk factors of these side effects (2). LID can be a very disabling and hard to treat side effect once it is established. It not only interferes with the patient's quality of life but also places a significant burden on the health care system. Knowledge of how to prevent the onset of dyskinesia and the optimum way of managing it once it occurs is an unmet need. Amantadine is, to date, the only US Food and Drug Administration approved anti-dyskinetic medication that does not compromise antiparkinsonian medication's benefit (1), but it typically loses its efficacy in an average of 8 months (3).

The degree of LID fluctuates throughout the day, so its documentation is often highly dependent on subjective opinions of patients, which makes it difficult to conduct a clinical trial targeting LID. An objective and quantifiable biomarker for LID is highly desired to monitor the effects of treatment as well as to identify those individuals at higher risk of developing LID.

*In vivo* functional brain imaging techniques such as PET and fMRI have great clinical utility in monitoring disease progression and response to treatment in patients. Previously, functional imaging has shown that therapeutic levels of levodopa normalizes aberrant regional brain metabolism in Parkinson's disease patients, in a manner which correlates with clinical improvement of motor symptoms (4, 5). Radiological studies in LID patients have revealed that LID is acutely triggered by large, transient increases in striatal dopamine release following Levodopa administration (6). Patients who do not have motor complications show stable levels of synaptic dopamine concentration after Levodopa administration (7–9). In comparison, at 4 h post Levodopa administration PD patients with motor complications have significant synaptic dopamine level reduction in the putamen, whereas in the stable group synaptic dopamine level remains constant (8, 9). These findings suggest that rapid swing and turnover of Levodopa levels at striatal synapses may contribute to the development of Levodopa—induced motor complications (9).

This acute overshoot of dopaminergic input after Levodopa administration can be partly explained by both the serotonergic reserve hypothesis and the neurovascular de-coupling hypothesis (10). The former suggests that Levodopa is converted to dopamine by reserved serotonergic neurons in the putamen then released in an uncontrolled manner due to lack of auto-receptors feedback mechanisms (11). The latter suggests that sustained D1 receptor stimulation resulting from the down-regulated reuptake (as part of a compensatory mechanism) may induce angiogenesis in the putamen (12–14). As dopamine also acts as a vasodilator (15), acute Levodopa challenge can increase cerebral blood flow while it normalizes pathologically high neuronal activity in the putamen, resulting in excessive levodopa delivery and dopamine release (16). This dissociation is a violation of the neurovascular coupling hypothesis, which is attributed to the potent vasoactive effects of levodopa on cerebral microvasculature through D1-like receptors on astrocytes and vascular smooth muscle (17–20). The neurovascular coupling hypothesis suggests that an increase neuronal activity is followed by increased cerebral metabolic rate (CMR) and cerebral blood flow (CBF) (21). The neurovascular “de-coupling” phenomenon is especially predominant in PD patients with LID (22, 23), suggesting that chronic Levodopa exposure induces angiogenesis and that it may be involved with LID (16). A previous study investigated the effect of constant Levodopa infusion (intravenous Levodopa infusion titrated to achieve maximal improvement in PD motor symptoms without inducing dyskinesia) and its related motor complications in PD (22). They used [^15^-O]-H_2_O PET to measure CBF and fluorodeoxyglucose (FDG)-PET to measure CMR, finding that CBF was increased while CMR was decreased by Levodopa in the putamen, thalamus, pons, and subthalamic nucleus (STN). Based on the hypothesis that the changes in FDG PET signal represents synaptic activity (24), the dissociation between CMR and CBF violates the neurovascular coupling hypothesis. This dissociation was especially predominant in PD patients with LID compared to those without.

Previous fMRI studies have indicated that cortical regions such as the supplementary motor area (SMA), primary motor cortex (M1), and right inferior frontal gyrus (rIFG) also play a key role in the development and severity of LID in PD patients (25–28). Dopaminergic modulation of cortico-striatal networks could therefore be used to classify LID and non-LID patients. A recent analysis using 6 LID and 6 non-LID subjects found that levodopa-induced reduction in resting state functional connectivity between the putamen and M1 predicted LID with 91% sensitivity and 100% specificity (29). Additionally, dopaminergic modulation of putamen-SMA connectivity was shown to negatively correlate with dyskinesia severity (30).

In the current study, we investigate if clinically determined anti-parkinsonian medications also dissociate the CBF and glucose metabolic activity using perfusion MRI (pMRI) and FDG-PET, respectively. Additionally, we investigate the proposed effect of anti-parkinsonian medications on cortico-striatal functional connectivity in LID and non-LID patients. We further propose an analytic method to quantify the degree of dissociation in the putamen which may have the potential to be used as a biomarker for LID.

## Materials and Methods

### Subjects

In order to investigate the potential effects of anti-parkinsonian medications, 10 PD patients (age: 67 ± 7.71, 8 males, 5 with LID, disease duration: 9.22 ± 4.54 years) have been recruited from a local movement disorder clinic in Winnipeg, Manitoba, Canada. All patients were on clinically determined anti-parkinsonian medications. Only patients who had been taking Levodopa for the last 3 months without any changes in dosage were recruited. Each patient was scanned by FDG-PET and functional MRI when she/he was on anti-parkinsonian medications (ON) vs. while all anti-parkinsonian medications were withdrawn for >12 h (OFF). For PET scans in the ON condition, patients took their regular morning dose of anti-parkinsonian medications before FDG injection, which was followed by PET scans 30–45 min later. For MRI, patients took their medications 30–45 min before the scan. The severity of clinical symptoms was evaluated immediately after the FDG-PET scans by the Movement Disorder Society—Unified Parkinson's Disease Rating Scale (MDS-UPDRS) (31) and LID severity was evaluated with the Abnormal Involuntary Movement Scale (AIMs) (32). Patients were also assessed using the Beck Depression Inventory (BDI-II) (33) and Montreal Cognitive Assessment (MoCA) (34). The study was approved by the Biomedical Research Ethics Board at the University of Manitoba, and written informed consent was obtained from each subject. Summary of demographic information of the study group is presented in Table 1.

**Table 1:** Summary of PD patients' demographic information in the current study.

**Group**	**ID**	**Age**	**Sex**	**PD duration (years)**	**MoCA**	**LID duration (years)**	**BDI**	**MDS-UPDRS-III**		**AIMS**	**Anti-PD medications**
								**OFF**	**ON**		
LID	PD02	76	M	6	22	2.5	12	31	19	2	LD. Amantadine
	PD03	66	M	11	23	1.25	6	20	10	1	LD. DA
	PD05	73	M	18	21	6	9	52	43	3	LD. Amantadine
	PD09	55	M	5	24	1.5	6	37	30	2	LD.
	PD10	65	F	14	29	8	10	37	21	3	LD. Amantadine
Non-LID	PD01	55	M	5	30		0	19	9	0	LD. Amantadine
	PD04	71	M	7	29		4	31	30	0	LD. DA.MOA-B.
	PD06	64	M	4	23		5	53	14	0	LD.
	PD07	68	M	8	27		8	22	16	0	LD.
	PD08	77	F	10	29		4	24	23	0	LD.MOA-B
*P*-value		1.0		0.22	0.07		0.13	0.32	0.49	0.004	

*PD, Parkinson's disease; MoCA, Montreal cognitive assessment test; MDS-UPDRS, movement disorder society—unified Parkinson's disease rating scale; AIMS, abnormal involuntary movement scale; LD, Levodopa; DA, dopamine agonist; MOA-B, Monoamine oxidase B inhibitors; M, male; F, female*.

### Image Acquisition

#### Magnetic Resonance Imaging

All patients underwent MRI using a 3T Siemens/IMRIS MR System equipped with an 18 channel head coil located at the Kleysen Institute for Advanced Medicine at the Bannatyne campus of the University of Manitoba. The structural T1-weighted image utilized the MP-RAGE pulse sequence with an acquisition time of 8 min. The CBF acquisition utilized the pseudo-continuous arterial spin labeling (pCASL) pulse sequence with an acquisition time of 5 min (35). Acquisition parameters were TR = 4.0 s, TE = 12 ms, FOV = 240 × 240 mm^2^, matrix = 64 × 64 × 20, slice thickness = 5 mm, inter-slice space = 1 mm, labeling time = 2 s, post label delay time = 1.2 s, bandwidth = 3 kHz/pixel, flip angle = 90°. Forty five label/control image pairs were acquired for each subject.

Each fMRI session was comprised of 300 T2^*^ weighted echo planar images (FOV 220 mm, slice thickness 4.0 mm, TR 2,000 ms, TE 28 ms, Flip Angle 77°, with 37 total slices covering the whole brain volume). All subjects underwent metabolic imaging with FDG PET after fasting for at least 6 h before scanning. Patients were injected i.v. with 185 MBq of FDG and a 15 min static image was acquired starting 40 min post-injection. A head CT scan was acquired for attenuation correction purposes. All PET imaging data for this project were acquired on a Siemens Biograph 16 HiRez PET/CT (Siemens Medical Solutions, Knoxville, TN) scanner located in the John Buhler Research Centre at the Bannatyne campus of the University of Manitoba.

### Image Pre-processing

Resting-state fMRI images were realigned to the first image in the set and head movement parameters were extracted and used as regressors of no interest in first level analysis. CBF images were derived from the pCASL images using the Arterial Spin Labeled Perfusion MRI data processing toolbox (35), then pre-processed using SPM12 (http://www.fil.ion.ucl.ac.uk/spm/software/spm12/). For both resting-state fMRI and CBF, the standard procedures, i.e., co-registration with structural T1-MRI, normalization to ICBM template, segmentation, and smoothing with 8 × 8 × 8 mm Gaussian kernel were performed with the default parameters. The same SPM pre-processing steps were performed on all FDG PET images with their corresponding structural T1-MRI using SPM12. Voxel values were divided by the mean value of white matter to account for non-specific inter-individual differences (36, 37).

### Neurovascular Uncoupling Analysis

Based on the previous study that demonstrated dissociation between CBF and CMR (22), multiple key subcortical brain regions of interest (ROI) were defined including: putamen, caudate, thalamus, M1, and STN as delineated in automated anatomical labeling (38). The mean CBF and FDG uptake values were extracted for each region under different conditions (ON and OFF scans).

### Putamen Hyper-Perfusion/Hypo-Metabolism Index (PHI)

**Figure 1:**
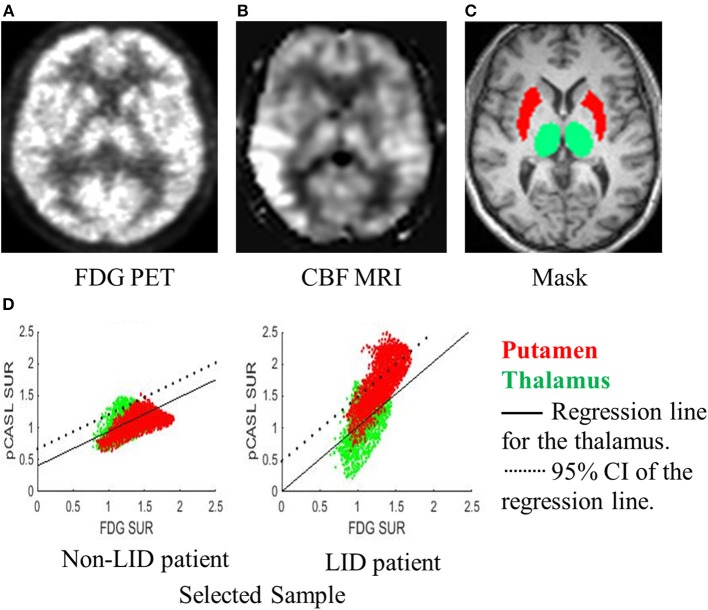
A novel method for quantifying dissociation of metabolic-blood flow with only ON-scans. A representative subject's **(A)** FDG-PET, **(B)** CBF-MRI, and **(C)** mask labeling are displayed. **(D)** Regression between FDG-PET and CBF-MRI was performed for the voxels in the thalamus (green dots). A voxel-to-voxel plot of FDG-PET (x-axis) and CBF-MRI (y-axis) and regression line (black solid line) and 95% confidence interval (CI; dotted line) for the thalamus are displayed for a sample patient without LID (left) and a patient with LID (right). The proportion of voxels in the putamen (red dots) falling above the 95% CI estimated from the thalamus was quantified and named the Putamen Hyper-perfusion/hypo-metabolism Index (PHI). The PHI score represents the spatial extent of voxels that are hyper-perfused compared to the thalamus.

**Figure 2:**
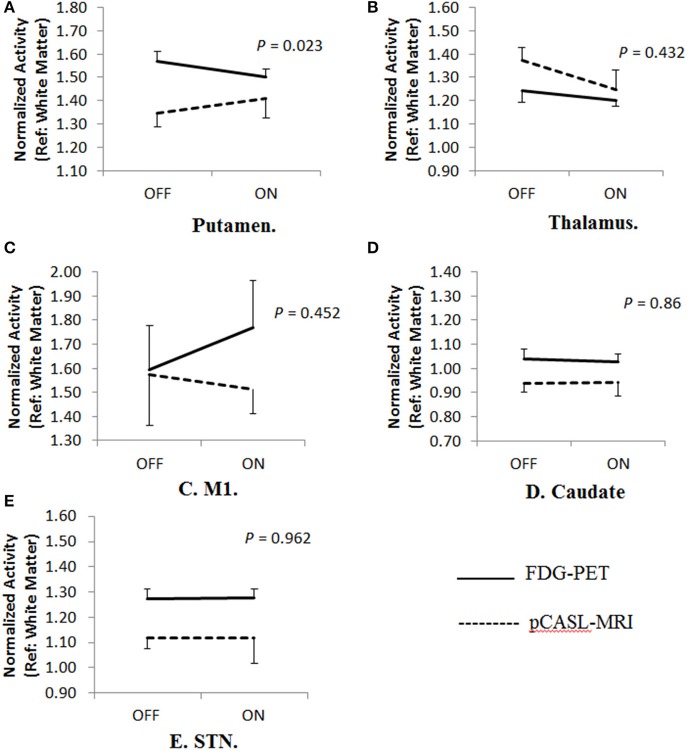
Cerebral blood flow-metabolism dissociation in different brain regions. **(A)** A significant Cerebral Blood Flow-Metabolism dissociation in the putamen using FDG-PET and pCASL-MRI [Interaction effects, *F*_(1,8)_ = 7.491, *p* = 0.023]. In the putamen, the FDG uptake was consistently decreased by anti-parkinsonian medication (*p* = 0.001, *post-hoc* Bonferroni) while mixed effects were observed in CBF changes (*p* = 0.214, *post-hoc* Bonferroni). When different groups are separately analyzed, trend-level of interaction effects were only observed in LID group [medication × modality: *F*_(1,4)_ = 5.648, *p* = 0.076] but not in non-LID group [medication × modality: *F*_(1,4)_ = 2.334, *p* = 0.201]. However, no significant dissociation was observed in other regions including **(B)** thalamus [*F*_(1,8)_ = 0.678, *p* = 0.432], **(C)** primary motor area (M1) [*F*_(1,8)_ = 0.618, *p* = 0.452], **(D)** caudate [*F*_(1,8)_ = 0.033, *p* = 0.86], and **(E)** subthalamic nucleus (STN) [*F*_(1,8)_ = 0.002, *p* = 0.962].

While the medication-induced flow-metabolism dissociation can be readily estimated by comparing the two conditions (ON vs. OFF), it may not be a practically desirable method that can be easily implemented in clinical or research trial settings. We developed a novel brain imaging-based method that quantifies the spatial extent of putamen hyper-perfusion/hypo-metabolism in which only the ON condition scans are used. The thalamus was selected as a reference region for following the reasons: (1) the neurovascular coupling hypothesis is not violated in the thalamus when estimated with FDG PET and pCASL MRI (**Figure 2B**); (2) the D1 receptor, i.e., the main pathway of Levodopa-induced vasodilation, is reported to be the least available in the thalamus (39), and (3) it is located in close proximity and has a similar size to the putamen, thus the image quality and spatial resolution is similar for the thalamus and the putamen. The Putamen Hyper-perfusion/Hypo-metabolism Index (PHI) was defined as the proportion of voxels in the putamen that lie above 95% CI of the regression line of the thalamus between FDG uptake vs. pCASL perfusion (Figure 1). Therefore, the null hypothesis is that the relationship between perfusion and glucose metabolism is linear and it should be identical between the putamen and the thalamus in the “normal” condition. If the degree of relative hyper-perfusion and hypo-metabolism in the putamen is beyond the 95% CI of the thalamus, it may be pathological. Thus, the PHI score represents the spatial extent of abnormally hyper-perfused and/or hypo-metabolic voxels in the putamen.

### Functional Connectivity Analysis

The first eigenvariate of all voxels for the left and right putamen for the timeseries of each subject was extracted and used as a regressor for a general linear model for each subject. This produced a statistical parametric map for each subject in which the beta weights represent the coefficient of covariance between that particular voxel and the putamen in that subject, representing a measure of functional connectivity. The regional coefficient of covariance was extracted from each ROI using the unthresholded first eigenvariate of that anatomical region. From this we generated the coefficient of connectivity between the seed region (bilateral putamen) and each ROI in our analysis in both the OFF state and ON state from each patient. The measures for each region were converted to z-scores by subtracting each connectivity coefficient from the group mean (including both LID and non-LID patients in the OFF state) and dividing by the standard deviation. Dopaminergic modulation of resting state connectivity was calculated as the difference in z-scores between the OFF and ON state for each patient.

### Statistical Analysis

The Student *t-*test was performed to investigate group differences in age, disease duration, PD motor symptoms severity, cognition impairment, and depression between LID vs. non-LID subjects. The neurovascular uncoupling results were analyzed by the 2 × 2 repeated measures ANOVA to investigate the main effect of imaging modality (PET vs. MRI) and medication (ON vs. OFF) and their interaction effects. When applicable, a *post-hoc* Bonferroni test was performed. The functional connectivity results (comparing LID vs. non-LID) were assessed via *t*-test. The applicability of PHI (ON) score for differentiating LID vs. non-LID was examined by observing how many patients were above the “normal” level of PHI score determined in the OFF condition (mean + 2SD).

## Results

### Clinical Effects of Anti-parkinsonian Treatment

Patients were not significantly different between the groups (LID vs. non-LID) in overall motor symptoms [MDS-UPDRS-III: (ON) *t*_(8)_ = 0.755, *p* = 0.492, (OFF) *t*_(8)_ = 1.116, *p* = 0.327], age [*t*_(8)_ = 0, *p* = 1.0], cognitive symptom severity [MoCA: *t*_(8)_ = 2.031, *p* = 0.077], depression level [BDI-II: *t*_(8)_ = 1.901, *p* = 0.130], and disease duration since the first diagnosis [*t*_(8)_ = 1.423, *p* = 0.228]. In 2 × 2 repeated measures ANOVA, all patient's motor symptoms were ameliorated by anti-parkinsonian medication [Effect of medication: *F*_(1, 8)_ = 9.334, *p* = 0.016] and no significant group difference was noted in changes in MDS-UPDRS-III (interaction effect of medication × group: *F*_(1, 8)_ = 0.007, *p* = 0.936]. As expected, non-LID patients do not show any signs of dyskinesia when assessed by AIMs. In LID patients, the severity of dyskinesia varied across patients (Table 1).

### Cerebral Blood Flow—FDG Uptake Dissociation Response to Anti-parkinsonian Treatment in Parkinson Patients

In the 2 × 2 repeated measures ANOVA, there were no significant main effects of different imaging modality (FDG-PET vs. pCASL-MRI: *p* > 0.075) or anti-parkinsonian medications (OFF vs. ON: *p* > 0.099) in any regions investigated. Significant interaction effect (medication × modality) has been only found in the putamen [*F*_(1, 8)_ = 7.491, *p* = 0.023], but not in the thalamus [*F*_(1, 8)_ = 0.678, *p* = 0.432], caudate [*F*_(1, 8)_ = 0.033, *p* = 0.860], STN [*F*_(1, 8)_ = 0.002, *p* = 0.962], nor M1 [*F*_(1, 8)_ = 0.618, *p* = 0.452] (Figure 2). In the putamen, the FDG uptake was consistently decreased by anti-parkinsonian medication (*p* = 0.001, *post-hoc* Bonferroni) while mixed effects were observed in CBF changes (*p* = 0.214, *post-hoc* Bonferroni). Interestingly, when different groups are separately analyzed, a trend-level of interaction effect was observed in the LID group [medication × modality: *F*_(1, 4)_ = 5.648, *p* = 0.076] but not in the non-LID group [medication × modality: *F*_(1, 4)_ = 2.334, *p* = 0.201].

### Dopaminergic Modulation of Resting State Connectivity

**Figure 3:**
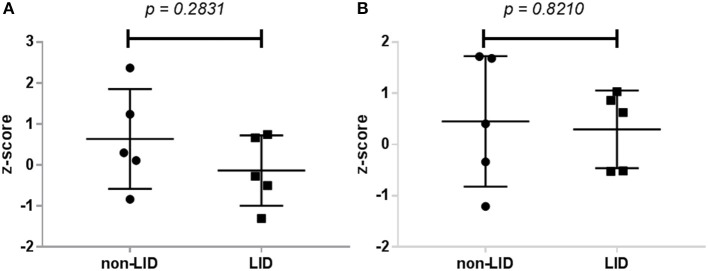
Dopaminergic modulation of resting-state connectivity in LID and non-LID patients. **(A)** Change in connectivity between bilateral putamen and M1. **(B)** Change in connectivity coefficient between putamen and SMA. Dopaminergic modulation was calculated as the difference in z-transformed connectivity coefficients between all voxels in the seed region (putamen) with the region of interest from the OFF condition to the ON condition.

No significant interaction effects of resting-state connectivity (group vs. medication) were noted in any of the ROIs examined, including between the bilateral putamen and M1 (*p* = 0.2831) or between bilateral putamen and SMA (*p* = 0.8210; Figure 3). Importantly, these results did not change when using ROIs from only the most affected hemisphere (ipsilateral to the side with greatest symptoms measured with UPRDS-III).

### Putamen-to-Thalamus Hyper-Perfusion/Hypo-Metabolism Index (PHI)

**Figure 4:**
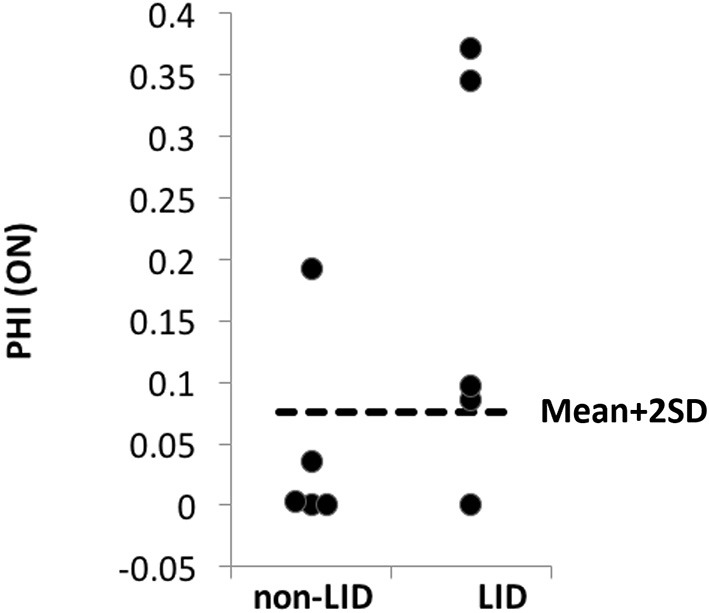
PHI comparison between LID and non-LID. The “normal” level of PHI was determined based on the scores of all patients (*n* = 10) estimated at the OFF condition assuming that no dissociation occurs in the OFF condition. Any PHI scores above mean + 2 × SD (dashed lines) were considered “abnormal.” In the ON condition, 4 out of 5 LID patients' PHI scores were abnormally high, while only 1 out of 5 non-LID patients' PHI score was higher than the normal range.

The PHI was introduced to estimate the spatial extent of relative hyper-perfusion and hypo-metabolism of the putamen compared to the thalamus, which has similar features (see section Materials and Methods). Anti-parkinsonian treatment increased PHI above the mean + 2SD (determined in the OFF condition) in 4 of 5 LID patients and 1 of 5 non-LID patients (Sensitivity = 0.8, Specificity = 0.8; Figure 4).

## Discussion

As expected based on prior studies (22, 23), putamen FDG uptake was reduced by clinically-determined anti-parkinsonian medications in all patients, suggesting that the therapy normalized the pathologically hyper-metabolic state of the putamen (16). However, its effect on CBF was not uniform, resulting in significant dissociation between CBF and FDG uptake. The separate group analysis suggests that the significant dissociation mainly originated from LID patients rather than non-LID patients, which is in line with the previous studies (22, 23).

Unlike in previous studies, dissociation in other brain regions (i.e., thalamus, STN, caudate, and M1) was not observed (22, 23). This discrepancy may have originated from the previous studies only investigating the effects of Levodopa while other anti-parkinsonian medications (e.g., dopamine agonist, monoamine oxidase B inhibitor) were also withheld for the OFF condition in our study. In addition, four patients were on amantadine [half-life: 9.7–14.5 h (40)] which has been shown to have anti-dyskinetic and vasoconstrictive effects (41). It should be noted that the LID patients' dyskinesia was not completely controlled by their current anti-parkinsonian/anti-dyskinetic treatment while in contrast, the previous studies used Levodopa infusion with dose titrated not to induce dyskinesia during scanning (22, 23). This signifies the relevance of the putamen's dissociation with LID over other brain regions that have been tested, which was most prominent among the regions that previously showed dissociation (23).

Different hypotheses lie behind CBF and FDG uptake dissociation. Experiments with 6-OHDA-lesioned rats have demonstrated Levodopa-mediated blood flow-metabolism dissociation in the striatum after both acute and chronic injection of Levodopa (42, 43). The acute rise in CBF is mediated by increases in the blood-brain-barrier (BBB) permeability as well as vasodilation controlled by smooth muscle cells and endothelium changes in the striatum (16). This dissociation is specific to the regions of dopaminergic degeneration and was not reported in the contralateral intact hemisphere of the 6-OHDA lesioned PD animals (12). Chronic exposure to Levodopa induced significant growth of immature endothelial cells, stimulated micro-vessel proliferation and increased synthesis and expression of vascular endothelial growth factor (VEGF) in the basal ganglia (14). The degree of VEGF expression was positively correlated with the total dose of Levodopa (14). A post-mortem study with PD patients also revealed significant VEGF transcriptions and subsequent expression of VEGF mRNA and an increase in nestin stain (a marker of immature endothelial cells) (43). Interestingly, in rat models following chronic exposure with Levodopa, VEGF expression, angiogenesis, and proliferating micro vessels stained by nestin were more prominent in animals with dyskinesia (14). These findings were reversed by a VEGF signaling inhibitor, which reduced dyskinesia in PD-LID model animals (43).

At therapeutic doses of Levodopa, dopamine has a vasodilation effect and increases regional CBF, which facilitates the transport of the drug across the BBB (15). The increased microvasculature discussed above may prime the regional neurovascular unit to have an exaggerated response to Levodopa-supplied dopamine transmission and further stimulate angiogenesis, forming a vicious cycle to further increase dopamine transmission beyond the optimal level (16).

As an alternative brain-imaging biomarker for LID, we investigated dopaminergic modulation of resting-state connectivity in the cortico-striatal axis using M1 and SMA as our primary ROI. This is based on the theory that abnormal dopaminergic modulation of the cortico-basal ganglia motor loops preconfigure the emergence of LID (44). We have not found a significant difference in dopaminergic modulation of resting state connectivity between LID and non-LID patients in either of these ROIs either bilaterally or using the most affected hemisphere. In a previously reported study dopamine treatment was shown to significantly decrease connectivity between putamen and M1 in LID patients compared to non-LID patients with a very high sensitivity (91%) and specificity (100%) (30). It is important to note that low sample size or differences in scanning protocol may have reduced the sensitivity of this technique in our subjects.

### The Use of PET+MRI as a LID Biomarker

Dopamine has a vasodilating effect in the putamen through stimulation of D1 receptors (17). It has been reported previously that D1 receptors are found with high density in the striatum, nucleus accumbens, and substantia nigra pars reticulata (18). However, the D1 receptor, i.e., the main pathway of Levodopa-induced vasodilation and angiogenesis (43), is reported to be the least available in the thalamus (39). This makes the thalamus the optimal reference region to estimate the CBF-FDG uptake dissociation (see section Materials and Methods).

We found that in 4 out of 5 LID patients the PHI score in the ON condition is above the normal level (mean + 2SD of the OFF condition), suggesting that it may be used as a sensitive biomarker for LID (80% sensitivity, albeit with a small sample size). Patient PD010 is the only LID patient whose PHI is below the abnormality threshold. It should be noted that multiple hypotheses exist surrounding the pathophysiology of LID (10, 45). Indeed, this patient's LID may not be related to Levodopa-induced angiogenesis/vasodilation but may exclusively involve other pathways such as serotonergic reserve (46, 47). This finding can be interpreted as suggesting that those patients who have low PHI score may not benefit from anti-angiogenic treatment, a potential preventive medicine against LID (48). Also, this type of patient (who shows low PHI score) may need to be precluded from future clinical trials and subsequent clinical practice targeting angiogenesis.

Interestingly, patient PD010 is also the only patient in the LID group who had depression and is treated with an atypical antidepressant, Bupropion, which is a dopamine norepinephrine reuptake inhibitor. This could interfere with neurotransmitter balance and inhibit regional CBF. Norepinephrine acts on alpha 1 and alpha 2 receptors in most systemic arteries and veins and induces vasoconstriction, which eventually raises the systemic vascular resistance and reduces blood flow (49, 50).

Among non-LID patients, only one patient (PD004) out of five lies above the abnormality threshold (mean + 2SD of the OFF condition). The adjusted *R*^2^ of the thalamic linear regression model of FDG uptake vs. CBF can serve as an indicator for nonspecific noise (e.g., motion artifacts and mis-registration) and it was the lowest in the PD004 (Adj. *R*^2^ = 0.0062; other image pairs' Adj. *R*^2^ > 0.14), suggesting a possibility of false positive due to technical issues rather than a physiological outlier. Potential implications for future research trials include that the patients in which the brain imaging pairs' Adj. *R*^2^ yields <0.1 may need to be re-scanned.

### Limitations

The power of statistical analysis is mainly defined by the sample size and noise level of the data. In this regard, the low sample size is the main limiting factor for this present study, which warrants a larger-scale longitudinal study to confirm the usability of the proposed method. Nevertheless, by utilizing the expected “normal” level of neurovascular coupling from the thalamus within each patient, the threshold for “abnormality” could be reliably estimated from the OFF condition, which reduced the non-specific noise associated with the image quality. PD is a movement disorder that affects non-medicated patients with tremor and medicated ones with potential dyskinesia, therefore it is typically very troublesome to ensure high quality imaging studies. Since the thalamus is located in a close proximity and has similar volume and spatial resolution as the putamen, it receives the same level of non-specific noise effects as the putamen, e.g., motion artifacts. The thalamic confidence level of the PET-MRI correspondence serves as an optimal indicator for defining “abnormality” in the putamen in the given imaging quality, which enhanced the statistical power of the proposed method.

The most interesting potential implication is whether the proposed PHI method can predict future emergence of LID from non-LID state, which can be only addressed with a longitudinal dataset. Our current study identified a non-LID patient (PD004) with a high PHI score, which warrants an on-going follow-up on dyskinesia state of this patient. Anecdotally, this patient has not developed LID in the 1 year since the PET+MRI scans. Moreover, we postulated that the high PHI score of this patient is likely due to a technical origin (Adj. *R*^2^ < 0.1), rather than a pathological origin.

## Conclusion

The exact pathology of LID in PD is not known. Using the flow-metabolism dissociation that is specific to LID, we proposed a novel PET+MRI-based biomarker for LID (i.e., PHI). Conditional on a larger-scale longitudinal study confirmation, the proposed method may be useful in identifying patients who are at risk of developing LID and who will most likely benefit from anti-angiogenic treatment, and in determining the outcome responses of a preventive medicine trial for LID.

## Data Availability Statement

The de-identified relevant data produced in the current study can be accessed via http://www.kolabneuro.com/. The raw data are not publicly available as they contain patient medical data which can only be accessed under the Personal Health Information Act (PHIA), information regarding which can be found at http://www.gov.mb.ca/health/phia/.

## Ethics Statement

The studies involving human participants were reviewed and approved by Biomedical Research Ethics Board, University of Manitoba. The patients/participants provided their written informed consent to participate in this study.

## Consent for Publication

All participating individuals gave an informed consent for us to publish the de-identified data that were collected from this study.

## Author Contributions

DH, LR, AG, and JK conceived the study. MA, DH, AB, KW, AK, LR, and AG contributed to the data collection including interviews, clinical tests, and imaging. MA, SB, and JK analyzed the data. MA and SB drafted the manuscript. All authors contributed to the revision of the manuscript.

### Conflict of Interest

The authors declare that the research was conducted in the absence of any commercial or financial relationships that could be construed as a potential conflict of interest.

## Abbreviations

AIMs: abnormal involuntary movement scale
BDI-II: beck depression inventory
BBB: blood-brain-barrier
CBF: cerebral blood flow
CMR: cerebral metabolic rate
FDG: fluorodeoxyglucose
fMRI: functional magnetic resonance imaging
LID: levodopa induced dyskinesia
MoCA: montreal cognitive assessment
MDS-UPDRS: movement disorder society—unified Parkinson's disease rating scale
PD: Parkinson's disease
pMRI: perfusion MRI
PET: positron emission tomography
M1: primary motor cortex
pCASL: pseudo-continuous arterial spin labeling
PHI: putamen hyper-perfusion/hypo-metabolism index
ROI: regions of interest
rIFG: right inferior frontal gyrus
STN: subthalamic nucleus
SMA: supplementary motor area
VEGF: vascular endothelial growth factor
LD: Levodopa.

## References

[1] Zesiewicz TA. Levodopa-induced Dyskinesia in Parkinson's disease: epidemiology, etiology, and treatment. Curr Neurol Neurosci Rep. (2007) 7:302. 10.1007/s11910-007-0046-y 17618536

[2] Aquino CC Fox SH. Clinical spectrum of levodopa-induced complications. Mov Disord. (2015) 30:80–89. 10.1002/mds.26125 25488260

[3] Thomas A Iacono D Luciano AL Armellino K Di Iorio A Onofrj M. Duration of amantadine benefit on dyskinesia of severe Parkinson's disease. J Neurol Neurosurg Psychiatry. (2004) 75:141–3. 14707325 PMC1757492

[4] Asanuma K Tang C Ma Y Dhawan V Mattis P Edwards C. Network modulation in the treatment of Parkinson's disease. Brain. (2006) 129:2667–78. 10.1093/brain/awl162 16844713 PMC4459513

[5] Feigin A Fukuda M Dhawan V Przedborski S Jackson–Lewis V Mentis MJ. Metabolic correlates of levodopa response in Parkinson's disease. Neurology. (2001) 57:2083–8. 10.1212/WNL.57.11.2083 11739830

[6] Pavese N Evans AH Tai YF Hotton G Brooks DJ Lees AJ. Clinical correlates of levodopa-induced dopamine release in Parkinson disease: a PET study. Neurology. (2006) 67:1612–7. 10.1212/01.wnl.0000242888.30755.5d 17101892

[7] De La Fuente-Fernández R Schulzer M Mak E Calne DB Stoessl AJ. Presynaptic mechanisms of motor fluctuations in Parkinson's disease: a probabilistic model. Brain. (2004) 127:888–99. 10.1093/brain/awh102 14960500

[8] De La Fuente-Fernández R Sossi V Huang Z Furtado S Lu JQ Calne DB. Levodopa-induced changes in synaptic dopamine levels increase with progression of Parkinson's disease: implications for dyskinesias. Brain. (2004) 127:2747–54. 10.1093/brain/awh290 15329355

[9] Niccolini F Loane C Politis M. Dyskinesias in Parkinson's disease: views from positron emission tomography studies. Eur J Neurol. (2014) 21:694-e43. 10.1111/ene.12362 24471508

[10] Angela Cenci M. Presynaptic mechanisms of L-DOPA-induced dyskinesia: the findings, the debate, the therapeutic implications. Front Neurol. (2014) 5:242. 10.3389/fneur.2014.00242 25566170 PMC4266027

[11] Politis M Wu K Loane C Brooks DJ Kiferle L Turkheimer FE. Serotonergic mechanisms responsible for levodopa-induced dyskinesias in Parkinson's disease patients. J Clin Invest. (2014) 124:1340–9. 10.1172/JCI71640 24531549 PMC3934188

[12] Lerner RP Bimpisidis Z Agorastos S Scherrer S Dewey SL Cenci MA. Dissociation of metabolic and hemodynamic levodopa responses in the 6-hydroxydopamine rat model. Neurobiol Dis. (2016) 96:31–7. 10.1016/j.nbd.2016.08.010 27544483 PMC5102795

[13] Troiano AR De La Fuente-Fernandez RJ Sossi VJ Schulzer MJ Mak EJ Ruth TJ. PET demonstrates reduced dopamine transporter expression in PD with dyskinesias. Neurology. (2009) 72:1211–6. 10.1212/01.wnl.0000338631.73211.56 19020294

[14] Westin JE Lindgren HS Gardi J Nyengaard JR Brundin P Mohapel P. Endothelial proliferation and increased blood – brain barrier permeability in the basal ganglia in a rat model of 3, 4-dihydroxyphenyl- L -alanine-induced dyskinesia. J Neurosci. (2006) 26:9448–61. 10.1523/JNEUROSCI.0944-06.2006 16971529 PMC6674611

[15] Iadecola C. Neurogenic control of the cerebral microcirculation: is dopamine minding the store? Nat Neurosci. (1998) 1:263–5. 10.1038/1074 10195155

[16] Ko JH Lerner RP Eidelberg D. Effects of levodopa on regional cerebral metabolism and blood flow. Mov Disord. (2015) 30:54–63. 10.1002/mds.26041 25296957 PMC4314428

[17] Choi J-K Chen YI Hamel E Jenkins BG. Brain hemodynamic changes mediated by dopamine receptors: Role of the cerebral microvasculature in dopamine-mediated neurovascular coupling. Neuroimage. (2006) 30:700–12. 10.1016/j.neuroimage.2005.10.029 16459104

[18] Chen YC Galpern WR Brownell AL Matthews RT Bogdanov M Isacson O. Detection of dopaminergic neurotransmitter activity using pharmacologic MRI: correlation with PET, microdialysis, and behavioral data. Magn Reson Med. (1997) 38:389–98. 10.1002/mrm.1910380306 9339439

[19] Chen Q Andersen AH Zhang Z Ovadia A Gash DM Avison MJ Mapping drug-induced changes in cerebral R2^*^ by Multiple Gradient Recalled Echo functional MRI. *Magn Reson Imaging*. (1996) 14:469–76. 10.1016/0730-725X(95)02100-8 8843359

[20] Sirohi D Chen Z Sun L Klose T Pierson TC Rossmann MG. The 3.8 Å resolution cryo-EM structure of Zika virus. Science. (2016) 352:467–70. 10.1126/science.aaf5316 27033547 PMC4845755

[21] Attwell D Buchan AM Charpak S Lauritzen M MacVicar BA Newman EA. Glial and neuronal control of brain blood flow. Nature. (2010) 468:232–43. 10.1038/nature09613 21068832 PMC3206737

[22] Hirano S Asanuma K Ma Y Tang C Feigin A Dhawan V. Dissociation of metabolic and neurovascular responses to levodopa in the treatment of Parkinson's disease. J Neurosci. (2008) 28:4201–9. 10.1523/JNEUROSCI.0582-08.2008 18417699 PMC2577921

[23] Jourdain VA Tang CC Holtbernd F Dresel C Choi YY Ma Y. Flow-metabolism dissociation in the pathogenesis of levodopa-induced dyskinesia. JCI Insight. (2016) 1:1–18. 10.1172/jci.insight.86615 27699242 PMC5033758

[24] Stoessl AJ. Glucose utilization: still in the synapse. Nat Neurosci. (2017) 20:382–4. 10.1038/nn.4513 28230843

[25] Cerasa A Koch G Donzuso G Mangone G Morelli M Brusa L. A network centred on the inferior frontal cortex is critically involved in levodopa-induced dyskinesias. Brain. (2015) 138:414–27. 10.1093/brain/awu329 25414038

[26] Cerasa A Morelli M Augimeri A Salsone M Novellino F Gioia MC. Prefrontal thickening in PD with levodopa-induced dyskinesias: New evidence from cortical thickness measurement. Park Relat Disord. (2013) 19:123–5. 10.1016/j.parkreldis.2012.06.003 22742954

[27] Cerasa A Pugliese P Messina D Morelli M Cecilia Gioia M Salsone M. Prefrontal alterations in Parkinson's disease with levodopa-induced dyskinesia during fMRI motor task. Mov Disord. (2012) 27:364–71. 10.1002/mds.24017 22076870

[28] Herz DM Haagensen BN Christensen MS Madsen KH Rowe JB L??kkegaard A Siebner HR. The acute brain response to levodopa heralds dyskinesias in Parkinson disease. Ann Neurol. (2014) 75:829–836. 10.1002/ana.24138 24889498 PMC4112717

[29] Herz DM Haagensen BN Christensen MS Madsen KH Rowe JB Løkkegaard A. Abnormal dopaminergic modulation of striato-cortical networks underlies levodopa-induced dyskinesias in humans. Brain. (2015) 138:1658–66. 10.1093/brain/awv096 25882651 PMC4614130

[30] Herz DM Haagensen BN Nielsen SH Madsen KH Løkkegaard A Siebner HR. Resting-state connectivity predicts levodopa-induced dyskinesias in Parkinson's disease. Mov Disord. (2016) 31:521–9. 10.1002/mds.26540 26954295 PMC5069605

[31] Ramaker C Marinus J Stiggelbout AM Van Hilten BJ. Systematic evaluation of rating scales for impairment and disability in Parkinson's disease. Mov Disord. (2002) 17:867–76. 10.1002/mds.10248 12360535

[32] Gharabawi GM Bossie CA Lasser RA Turkoz I Rodriguez S Chouinard G. Abnormal Involuntary Movement Scale (AIMS) and Extrapyramidal Symptom Rating Scale (ESRS): cross-scale comparison in assessing tardive dyskinesia. Schizophr Res. (2005) 77:119–28. 10.1016/j.schres.2005.03.008 15913963

[33] Beck AT Steer RA Ball R Ranieri W. Comparison of beck depression inventories -IA and -II in psychiatric outpatients. J Pers Assess. (1996) 67:588–97. 8991972 10.1207/s15327752jpa6703_13

[34] Nasreddine ZS Phillips NA Bédirian V Charbonneau S Whitehead V Collin I. The montreal cognitive assessment, MoCA: a brief screening tool for mild cognitive impairment. J Am Geriatr Soc. (2005) 53:695–9. 10.1111/j.1532-5415.2005.53221.x 15817019

[35] Wang Z Aguirre GK Rao H Wang J Fernández-Seara MA Childress AR. Empirical optimization of ASL data analysis using an ASL data processing toolbox: ASLtbx. Magn Reson Imaging. (2008) 26:261–9. 10.1016/j.mri.2007.07.003 17826940 PMC2268990

[36] Borghammer P Jonsdottir KY Cumming P Ostergaard K Vang K Ashkanian M. Normalization in PET group comparison studies—The importance of a valid reference region. Neuroimage. (2008) 40:529–40. 10.1016/j.neuroimage.2007.12.057 18258457

[37] Spence JS Carmack PS Gunst RF Schucany WR Woodward WA Haley RW. Using a white matter reference to remove the dependency of global signal on experimental conditions in SPECT analyses. Neuroimage. (2006) 32:49–53. 10.1016/j.neuroimage.2006.03.025 16651010

[38] Tzourio-Mazoyer N Landeau B Papathanassiou D Crivello F Etard O Delcroix N. Automated anatomical labeling of activations in SPM using a macroscopic anatomical parcellation of the MNI MRI single-subject brain. Neuroimage. (2002) 15:273–89. 10.1006/nimg.2001.0978 11771995

[39] Abi-Dargham A Moore H. Prefrontal DA transmission at D1 receptors and the pathology of schizophrenia. Neuroscience. (2003) 9:404–16. 10.1177/1073858403252674 14580124

[40] Horadam VW Sharp JG Smilack JD McAnalley BH Garriott JC Stephens MK. Pharmacokinetics of amantadine hydrochloride in subjects with normal and impaired renal function. Ann Intern Med. (1981) 94:454. 10.7326/0003-4819-94-4-454 7212501

[41] Demaagd G Philip A. Parkinson's disease and its management: part 3: nondopaminergic and nonpharmacological treatment options. P T. (2015) 40:668–9. 26535023 PMC4606857

[42] Aron AR Robbins TW Poldrack RA. Inhibition and the right inferior frontal cortex: one decade on. Trends Cogn Sci. (2014) 18:177–85. 10.1016/j.tics.2013.12.003 24440116

[43] Ohlin KE Sebastianutto I Adkins CE Lundblad C Lockman PR Cenci MA. Impact of L-DOPA treatment on regional cerebral blood flow and metabolism in the basal ganglia in a rat model of Parkinson's disease. Neuroimage. (2012) 61:228–39. 10.1016/j.neuroimage.2012.02.066 22406356 PMC4455552

[44] Calabresi P Picconi B Tozzi A Di Filippo M. Dopamine-mediated regulation of corticostriatal synaptic plasticity. Trends Neurosci. (2007) 30:211–9. 10.1016/j.tins.2007.03.001 17367873

[45] Iravani M Jenner P. Mechanisms underlying the onset and expression of levodopa-induced dyskinesia and their pharmacological manipulation. J Neural Transm. (2011) 118:1661–90. 10.1007/s00702-011-0698-2 21881839

[46] Hong JY Oh JS Lee I Sunwoo MK Ham JH Lee JE. Presynaptic dopamine depletion predicts levodopa-induced dyskinesia in de novo Parkinson disease. Neurology. (2014) 82:1597–604. 10.1212/WNL.0000000000000385 24719485

[47] Roussakis A-A Politis M Towey D Piccini P. Serotonin-to-dopamine transporter ratios in Parkinson disease: relevance for dyskinesias. Neurology. (2016) 86:1152–8. 10.1212/WNL.0000000000002494 26920358

[48] Ohlin KE Francardo V Lindgren HS Sillivan SE O'Sullivan SS Luksik AS. Vascular endothelial growth factor is upregulated by l-dopa in the parkinsonian brain: implications for the development of dyskinesia. Brain. (2011) 134:2339–57. 10.1093/brain/awr165 21771855 PMC3155708

[49] Hjemdahl P Belfrage E Daleskog M. Vascular and metabolic effects of circulating epinephrine and norepinephrine. Concentration-effect study in dogs. J Clin Invest. (1979) 64:1221–8. 10.1172/JCI109576 227927 PMC371267

[50] Quisth V Enoksson S Blaak E Hagström-Toft E Arner P Bolinder J. Major differences in noradrenaline action on lipolysis and blood flow rates in skeletal muscle and adipose tissue *in vivo*. Diabetologia. (2005) 48:946–53. 10.1007/s00125-005-1708-4 15778861

